# The impact of facility audits, evaluation reports and incentives on motivation and supply management among family planning service providers: an interventional study in two districts in Maputo Province, Mozambique

**DOI:** 10.1186/s12913-017-2222-3

**Published:** 2017-05-02

**Authors:** Heleen Vermandere, Anna Galle, Sally Griffin, Málica de Melo, Lino Machaieie, Dirk Van Braeckel, Olivier Degomme

**Affiliations:** 1 International Centre for Reproductive Health, Ghent University, De Pintelaan 185, Post: UZP114, 9000 Gent, Belgium; 2 International Centre for Reproductive Health - Mozambique, Rua das Flores no 34, Impasse 1085/87, Maputo, Mozambique

**Keywords:** Family planning services, Stock-outs, Motivation, Health care providers, Contraceptives, Mozambique, Incentives, Supply management

## Abstract

**Backrgound:**

Good progress is being made towards universal access to contraceptives, however stock-outs still jeopardize progress. A seldom considered but important building block in optimizing supply management is the degree to which health workers feel motivated and responsible for monitoring supply. We explored how and to what extent motivation can be improved, and the impact this can have on avoiding stock-outs.

**Methods:**

Fifteen health facilities in Maputo Province, Mozambique, were divided into 3 groups (2 intervention groups and 1 control), and 10 monthly audits were implemented in each of these 15 facilities to collect data through examination of stock cards and stock-counts of 6 contraceptives. Based on these audits, the 2 intervention groups received a monthly evaluation report reflecting the quality of their supply management. One of these 2 groups was also awarded material incentives conditional on their performance. A Wilcoxon-Mann Whitney test was used to detect differences between the groups in the average number of stocked-out centres, while changes over time were verified through applying a Friedman test. Additionally, staff motivation was measured through interviewing health care providers of all centres at baseline, and after 5 and 10 months. To detect differences between the groups and changes over time, a Kruskal Wallis and a Wilcoxon signed-rank test were applied, respectively.

**Results:**

Motivation reported by providers (*n* = 55, *n* = 40 and *n* = 39 at baseline, 1st and 2nd follow-up respectively) was high in all groups, during all rounds, and did not change over time. Facilities in the intervention groups had better supply management results (including less stock-outs) during the entire intervention period compared with those in the control group, but the difference was only significant for the group receiving both material incentives and a monthly evaluation. However, our data also suggest that supply management also improved in control facilities, receiving only a monthly audit. During this study, more stock-outs occurred for family planning methods with lower demand, but the number of stock-outs per family planning method in the intervention groups was only significantly lower, compared with the control group, for female condoms.

**Conclusions:**

While a rise in motivation was not measurable, stock management was enhanced possibly as a result of the monthly audits. This activity was primarily for data collection, but was described as motivating and supportive, indicating the importance of feedback on health workers’ accomplishments. More research is needed to quantify the additional impact of the interventions (distribution of evaluation reports and material incentives) on staff motivation and supply management. Special attention should be paid to supply management of less frequently used contraceptive methods.

**Electronic supplementary material:**

The online version of this article (doi:10.1186/s12913-017-2222-3) contains supplementary material, which is available to authorized users.

## Background

### Low uptake of contraceptives in Mozambique

Major efforts have been made in sub-Saharan Africa to improve access to contraception and to raise awareness about the benefits of family planning. Progress has been made, but contraception prevalence rates remain low and unmet need remains high. In 2015, almost 1 in 4 women (23.1%) in Mozambique reported an unmet need for family planning. The national Total Fertility Rate was 5.3 children per woman and the total modern contraceptive prevalence rate was only 25.3% (21.5% in rural areas and 34.3% in urban areas). This low coverage can be explained by a number of factors such as lack of knowledge and awareness, and socio-cultural factors [1]. Furthermore, various factors contribute to poor quality of service provision, including understaffing, health care workers who often lack skills, support and commitment, lack of equipment, and sub-optimal supply management leading to stock-outs of contraceptives [2, 3]. This situation entails a high risk of ill-informed choice of contraceptive methods, improper use and discontinuation.

### Supply chain management

In Mozambique, contraceptives – stored at regional, provincial and district warehouses - are distributed monthly under a “pull” system where primary health facilities request supplies from the district warehouse. In addition, emergency requests can be made in case of impending stock-outs; facilities are supposed to have a buffer stock for 1 month. At the facility level, pharmacists receive and store the contraceptives using stock-cards to maintain the inventory.

There are various problems throughout the supply chain, however, a large part of these are at a local level [4], including transportation delays, requisitions not fully supplied or inaccurate and/or delayed orders. Measures to improve family planning service delivery, including stock management of contraceptives, often tackle logistic and financial barriers, and build capacity of providers. Currently, supervision visits are foreseen from district to health facility level every 6 months, but also their implementation is hampered as they happen less frequently and irregularly and do not usually include stock audits (due to limited human and financial resources). Another factor that is seldom considered in efforts to strengthen supply chains is the role of motivation of health care workers.

### Motivation of health care workers

Poor motivation of health workers has been identified as an important problem by the Mozambican Ministry of Health [5] and also WHO highlights this issue in its 2006 report on the human resources crisis in health [6]. In addition, many researchers have suggested an important link between health worker motivation and quality of services [7–13], indicating that improving motivation could be the key to leaping to a much higher level of quality. Moreover, low motivation is not only likely to impact the quality of services directly, but also indirectly, for example through increasing staff turnover and absenteeism.

While salaries and benefits are generally considered as key determinants of (de)motivation, non-financial incentives also play an important role [10, 12]. When health care workers perceive that their professional needs are being accommodated (e.g. adequate equipment) or that someone is willing to invest in them (e.g. training and development), they are likely to reciprocate with improved performance [14]. This is confirmed by various studies: In Mali, for example, health workers reported that their main motivators were related to responsibility, training and recognition, as well as salary [15]. Health care providers in Adventist health facilities in Malawi on the other hand, found spiritual nourishment and working conditions with long term benefits motivating [16]. And finally, a study among health workers in Benin and Kenya also identified non-financial incentives and human resource management tools as important factors with respect to increasing health care providers’ motivation [10].

Besides merely identifying and recognizing the importance of non-financial incentives, interventional studies have also actually tested the impact of non-financial incentives on motivation and quality of care. For example, in Uganda, better job satisfaction was observed as an indirect measure of provider motivation, after implementing the ’Yellow Star Programme’ which was based on the possibility for providers to obtain awards linked to improving quality of care [17]. However, few studies have aimed to increase the motivation of health care providers involved in family planning services and supply management, especially in sub-Sahara Africa. Innovative methods are urgently needed to improve family planning services, including supply chains, and motivation of health workers is a factor that could potentially help [4].

### Objectives

We designed and implemented interventions to influence both staff motivation and supply management, in order to investigate the role of motivation in ensuring quality of family planning service, more specifically in monitoring stock and avoiding stockouts of contraceptives.

## Methods

### The interventions

#### The study context

Two districts in Maputo province, Mozambique, were included in the study (Manhiça and Marracuene). Of the 21 health centres in these districts, 15 were selected for this study and were randomly allocated into 3 groups (5 health centres in each group): 2 intervention groups and a third group as control. The remaining 6 centres were excluded due to being closely linked to larger health facilities, not offering family planning services, or being extremely hard to reach.

#### Monthly evaluation on supply management through awarding credits

**Fig. 1:**
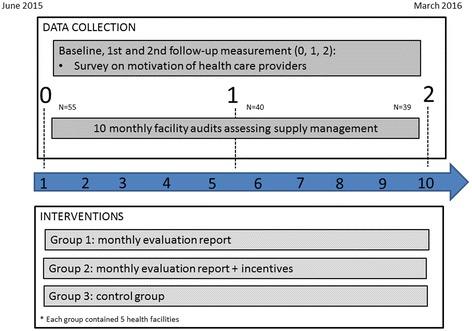
Project overview

In order to increase the motivation of the health care providers, an evaluation system was rolled out among the 10 health centres in intervention groups 1 and 2 (Fig. 1). This intervention aimed to reward health facilities for good supply management, through awarding credits to health centres based on their monthly performance. Each month health centres could earn a maximum of 3 credits by i) having stock cards of the 6 family planning methods (the combined contraceptive pill (Microgynon®), the low-dose contraceptive pill (Microlut®), the injectable contraceptive (Depo-Provera®), implant, an intra-uterine device (IUD) and female condom), ii) filling them in correctly (no calculation mistakes), and iii) reporting no stock-outs for any of the methods. The system was punitive for missing a stock card since without a card there was no control in terms of calculation mistakes or stock-outs, meaning that 1 missing stock card immediately led to 0 credits. Hence, health centres were encouraged to obtain a stock card for each of the 6 family planning methods, an essential first step of good supply management. Similarly, a stock card with a calculation mistake was considered unreliable meaning that the credit for not having a stock-out could not be earned.

Each month, health centres in groups 1 and 2 received a report with their results from the previous month so as to motivate them to improve their supply management or maintain good performance (Table 1). While health centres in group 3 did not receive this report, we did calculate their results as to compare their performance with the intervention groups. Before the start of the programme, staff from each health facility was trained in filling in the stock cards.

**Table 1:** Example of the credit system used to evaluate supply management in health centres

	Female condom	Implant	IUD	Injectable	Combined pill	Low-dose pil	Credits
Stock card	x	x	x	x	x	x	1
Calculation error	0	0	0	x	0	0	0
Reported stock-out	0	0	0	-	0	0	0
Total							1

x stands either for the presence of a stock card (leading to 1 credit in that category when all 6 stock cards were present in the facility), or for a calculation error or stock-out reported on it. In this example a calculation mistake on the stock card of Injectable led to 0 credits for that category since errors were punished. Similarly, the credit for not having a stock-out was not assigned, not because a stock-out was reported, in this example, but because the information was considered unreliable due to the calculation mistake (−). 0 stands for either no calculation mistake or stock-out reported on a card, or for 0 credits obtained for a category

#### Incentives

Besides being awarded credits and obtaining a report, the 5 health centres in group 2 could use the credits earned to buy small items for the facility (Fig. 1), such as small items of furniture and medical equipment or material. The more expensive the asset, the more credits were needed.

### Data collection

#### Monthly facility audits

In order to evaluate the health centres each month in function of the first intervention (i.e. awarding credits), monthly field visits were carried out during 10 months (Fig. 1). The 5 health centres of the control group were also visited to compare their results with those of the intervention groups. During these facility audits, photos were taken of the stock cards of the 6 family planning methods, if the cards were available, and field workers also counted the actual stock.

#### Survey on motivation

The motivation of health care providers of all 15 health centres was measured by a 23-question tool, developed by Mutale et al. [7], including 7 outcomes of motivation: i) general motivation; ii) burnout; iii) job satisfaction; iv) intrinsic job satisfaction; v) organisational commitment; vi) conscientiousness; and vii) timeliness and attendance. Participants had to agree or disagree with the statements through a 5-point Likert scale.

A cohort of health care workers was set up to measure motivation 3 times through a face-to-face questionnaire, at baseline, and after 5 and 10 months (Fig. 1). Through this we aimed at identifying changes in motivation over time as an effect of the interventions described above.

### Recruitment of participants

The project was explained to the heads of the 15 health centres, who were asked to inform their staff about the upcoming project. Afterwards, each of the health centres was visited and the project was again explained, this time to the entire staff. At that point, health care providers received general information regarding the monthly facility audits and the surveys on motivation. Each of the providers was then asked personally whether he or she agreed to participate in the project. In each of the health centres, field workers tried to include minimum 3 and maximum 5 providers for the motivation surveys, selecting those directly involved in family planning service provision and contraceptive stock management. Written consent was obtained from participants, which was later orally confirmed before the 1st and 2nd follow-up interview.

### Data analysis

#### Monthly facility audits

The data of the stock cards were entered in Microsoft Access and transferred to Stata13 for analysis. The number of available stock cards, and calculation errors and stock-outs identified on the stock cards were counted for each consecutive month. Results are expressed as percentages, i.e. the percentage of cards available out of the 6 that were requested each month, the percentage of available stock cards with 1 or more calculation errors, and the percentage of stock cards with 1 or more reported stock-out in the last 4 weeks.

When stock cards were not available in a health centre, 2 different analyses were conducted. In a first analysis, missing stock cards were considered as missing data with regard to calculation mistakes or reported stock-outs (resulting in a decreased denominator). The results of this analysis represent ‘lowest estimations’ since not having a stock card does not lead to any repercussion. This approach also favours centres with less stock cards given that they have simply less chance to make a calculation error or report a stock-out. In a second analysis, ‘upper estimations’ are presented: when a stock card is not available, we automatically penalized for calculation mistakes and for the presence of stock-outs (with the denominator remaining the same, i.e. 6 stock cards per health centre). Wilcoxon Mann–Whitney tests were applied to compare baseline results between the intervention groups and the control group, as well as the average number of credits earned over the 10 months in each of the groups. A Friedman test, generating Kendall’s coefficient, was used to detect changes in credits earned in the intervention period in all health centres and per group.

Finally, the data from the stock-counts were entered in Epi-info and, after transferring, analysed in Stata13. The number of stock-outs of the 6 contraceptives over the 10 months was calculated by health centre and by group. The information was also analysed by calculating the percentage of health facilities stocked out, by contraceptive method, on the day of assessment as well as averaged over the 10-month intervention period [18]. Using the Wilcoxon Mann–Whitney test, the monthly average of the sum of the stock-outs and the number of health facilities stocked-out for each contraceptive method separately was compared between the groups.

#### Survey on motivation

Data were entered in Epi-info 7 and transferred, through Microsoft Access, to Stata13. Negative statements were coded in the opposite direction, as such higher scores always indicate higher motivation. The score of each motivational outcome, i.e. the sum of the scores of the individual questions, was brought back to a scale of 1 to 5 in order to facilitate comparisons.

In a first step, baseline motivation of respondents and non-respondents (i.e. those lost at follow-up) was compared by a Wilcoxon-Mann Whitney test to verify whether or not respondents differed from non-respondents. A Monte Carlo simulation was used to define the confidence interval of the p-values. In a next step, differences between groups 1, 2 and 3 were identified, using a Kruskal Wallis test to compare the 3 groups. Finally, in order to detect changes over time, a Wilcoxon signed rank test was applied to compare baseline results with results of the first and second follow-up round. All statistical tests were performed at the 0.05 significance level.

## Results

### Participation

All 15 health centres agreed to participate in the study. At baseline (June 2015), 55 health workers were interviewed, and during the first (October 2015) and second follow-up (March 2016), motivation was measured again among 40 and 39 providers respectively. The number of participants at baseline, first and second follow-up were respectively 17, 12 and 10 for group 1, and 16, 12 and 12 for group 2, and 22, 16 and 17 for group 3.

### Motivation

#### Motivation at baseline

While the questionnaire on motivational outcomes contained 23 questions, 2 questions were deleted due to misinterpretations and errors in translation from English to Portuguese (both measuring conscientiousness). Baseline reported motivation was very high with a median score of 88.5/105. Answers of respondents who participated in the three rounds, and non-respondents were similar for all questions except for ‘intrinsic motivation’, which was slightly lower among those lost in follow-up (Table 2).

**Table 2:** Baseline motivation - comparison of respondents and non-respondents

Baseline motivation providers	All baseline participants (*n* = 55)	Respondents retained up to 2nd follow-up(*n* = 39)	Respondents lost in 1st or 2nd follow-up(*n* = 16)	Wilcoxon Mann–Whitneywith Monte-Carlo simulation
	Median (IQR)	Median (IQR)	Median (IQR)	*p*-value (CI)
Overall motivation (max 105)	88.5 (80–93)	89 (84–94)	85.5 (76–90)	0.13 (0.13–0.14)
General motivation	3.7 (3.3–4.3)	3.7 (3.3–4.3)	3.7 (3.3–4.3)	0.61
I feel motivated to work hard	4 (3–5)	4 (3–5)	4 (3–5)	0.79 (0.79–0.81)
I only do this job to get paid^a^	5 (4–5)	5 (4–5)	4 (4–5)	0.41 (0.40–0.42)
I do this job to have long-term security	3 (2–4)	3 (2–4)	3 (2–4)	0.81 (0.83–0.85)
Burn out (reversed)	3.5 (3.0–4.5)	3.5 (3.0–4.5)	3.7 (3.0–4.5)	0.53
I feel emotionally drained at end of day^a^	4 (2–4)	4 (2–4)	4 (4–4)	0.38 (0.38–0.40)
At times, I dread facing a day at work^a^	4 (2–5)	4 (2–5)	4 (2–5)	0.89 (0.92–0.93)
Job satisfaction	4.3 (3.7–5.0)	4.3 (4.0–5.0)	4.3 (3.5–4.7)	0.55
Overall, I am very satisfied with my job	5 (4–5)	5 (4–5)	5 (4–5)	0.31 (0.33–0.35)
I am not satisfied with my colleagues^a^	4 (4–5)	4 (4–5)	4 (4–4.5)	0.66 (0.65–0.67)
I am satisfied with my supervisor	4 (4–5)	4 (4–5)	4 (3.5–5)	0.37 (0.37–0.39)
Intrinsic motivation	4.3 (4.0–5.0)	4.7 (4.0–5.0)	4.0 (3.5–4.5)	0.01
Satisfied with opportunity to use abilities	5 (4–5)	5 (4–5)	5 (4–5)	0.27 (0.34–0.36)
Satisfied with accomplishing something	5 (4–5)	5(4–5)	4 (4–4.5)	0.01 (0.00–0.01)
My work is not valuable these days^a^	4 (4–5)	4 (4–5)	4 (2–4)	0.05 (0.05–0.06)
Organizational commitment	4.1 (3.6–4.6)	4.2 (3.6–4.6)	4.0 (3.4–4.4)	0.39
I am proud to work for this health facility	4 (4–5)	4 (4–5)	4 (4–5)	0.83 (0.88–0.89)
My values and this facility’s are similar	4 (4–5)	4 (4–5)	4 (3.5–4)	0.24 (0.23–0.25)
I am glad to work for this facility	4 (2–4)	4 (2–5)	4 (2–4)	0.41 (0.40–0.42)
I feel little commitment to this facility^a^	4 (4–5)	4 (4–5)	4 (2.5–4)	0.15 (0.14–0.15)
This facility inspires me to do my best	4 (4–5)	5 (4–5)	4 (4–5)	0.76 (0.79–0.80)
Conscientiousness	5 (4.5–5.0)	5 (4.5–5.0)	4.7 (4.5–5.0)	0.53
I am a hard worker	5 (4–5)	5 (4–5)	5 (4–5)	0.74 (0.76–0.78)
I do things without being asked or told	5 (4–5)	5 (5–5)	5 (4–5)	0.31 (0.33–0.35)
Timeliness and attendance	4.3 (4.0–5.0)	4.7 (4.0–5.0)	4.3 (4.0–5.0)	0.92
I am punctual about coming to work	5 (4–5)	5 (4–5)	4.5 (4–5)	0.93 (0.99–0.99)
I am often absent from work	5 (4–5)	5 (4–5)	4 (4–5)	0.36 (0.37–0.39)
Not a problem if I sometimes come late^a^	5 (4–5)	5 (4–5)	4 (4–5)	0.54 (0.51–0.53)

^a^reversed: a high score shows disagreement with a negative statement and is therefore suggestive of higher motivation

#### Changes in motivation

At baseline, first and second follow-up, no difference was found between the groups, either in overall motivation or in the subcomponents (Additional file 1). With regard to changes in motivation over time, no significant differences were detected comparing baseline with 1st and 2nd follow-up for each group (Table 3), or among all providers together (Additional file 2).

**Table 3:** Comparing baseline motivation with motivation reported at 1st and 2nd follow-up (*N* = 39)

	Baseline	1st Follow-up	2nd Follow-up	Wilcoxon signed ranks test (baseline – 1st follow-up)	Wilcoxon signed ranks test (baseline – 2nd follow-up)
	Median (IQR)	Median (IQR)	Median (IQR)	*p*-value (n)	*p*-value (n)
Group 1	88.5 (87–92)	87 (83–88)	90 (88–90)	0.66 (12)	0.68 (10)
Group 2	84.5 (79–93)	90 (86–93)	87 (83–90)	0.20 (10)	0.62 (11)
Group 3	93 (86.5–95)	86 (83–91)	87 (83–90)	0.28 (15)	0.38 (16)

### Supply management

#### Stock monitoring at baseline

At baseline, out of the 90 stock cards that were requested from the health centres (6 stock cards in each of the 15 facilities), 73 were available. The stock cards for female condoms were missing in 8 health centres, while the cards for IUDs, implants and the combined contraceptive pill were missing in 5, 3 and 1 health centres respectively (data not shown).

**Fig. 2:**
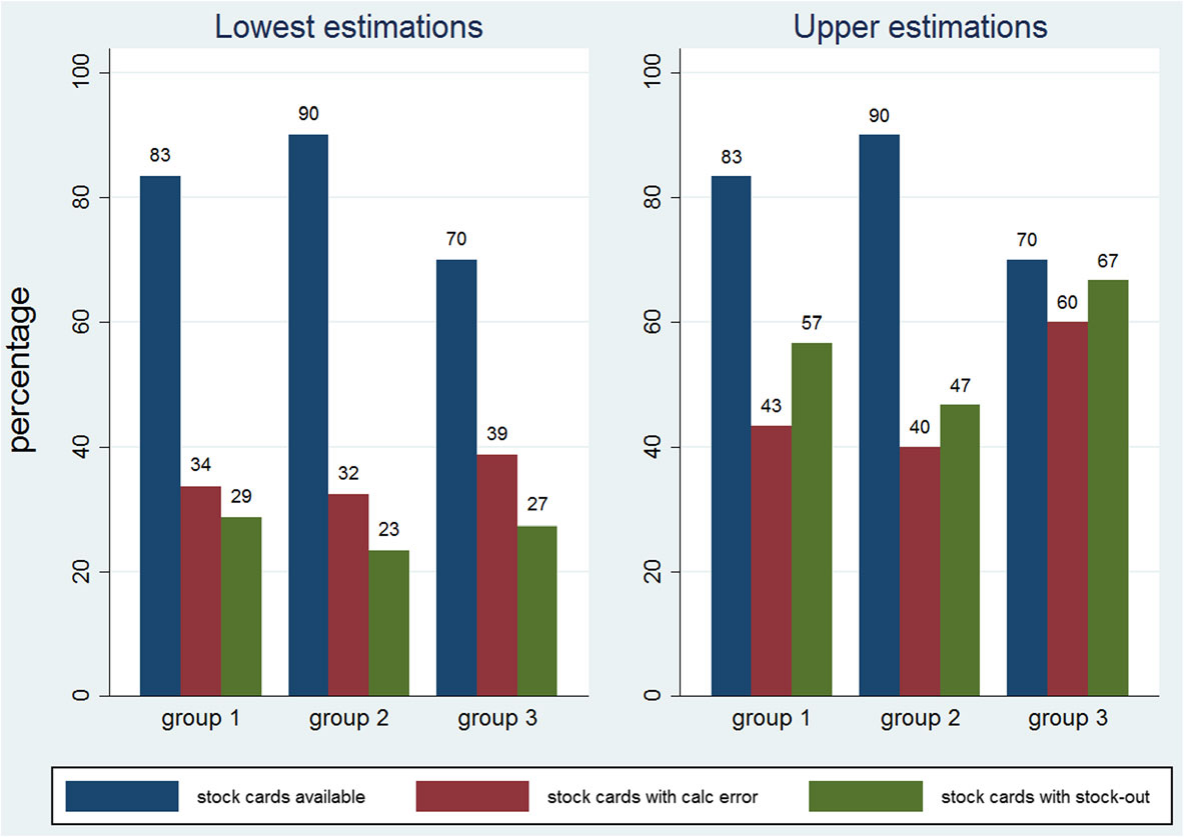
Baseline supply management. At baseline, out of the 30 stock cards requested in each group (5X6), 25, 27 and 21 stock cards were presented in group 1, 2 and 3, respectively. Calculation mistakes were found on 8 (1/4, 2/4, 3/5, 1/6 and 1/6), 9 (1/4, 1/5, 1/6, 3/6 and 3/6) and 9 (0/2, 2/4, 2/4, 3/5 and 2/6) of the cards in group 1, 2 and 3 (lower estimations), leading to 13/30, 12/30 and 18/30 mistakes according to the upper estimations in these groups, respectively. Similarly, stock were reported on 8 (0/4, 0/4, 3/5, 2/6 and 3/6), 6 (2/4, 0/5, 0/6, 2/6 and 2/6) and 5 (1/2, 0/4, 2/4, 1/5 and 1/6) of the cards in group 1, 2 and 3 (lower estimations), leading to 17/30, 14/30 and 20/30 stock-outs according to the upper estimations in these groups, respectively

The 3 groups were comparable, with the 3rd group, i.e. the control group, having slightly poorer descriptive results. As can be seen in Fig. 2, group 3 has on average less stock cards (70%, versus 83% and 90% in groups 1 and 2 respectively), and more stock cards had a calculation error. As a consequence, the ‘upper estimations’ of calculation mistakes and reported stock-outs were considerably higher for group 3, since in that analysis a missing stock card and/or a calculation mistake were considered as a stock-out. However, none of the supply management scores – credits, number of stock cards, and upper and lower estimations of calculation errors and stock-outs - were significantly different for the intervention groups and the control group at baseline (except for the upper estimation of calculation mistakes comparing group 2 and group 3, with group 3 having more mistakes; *p* = 0.05).

#### Changes in stock monitoring

**Fig. 3:**
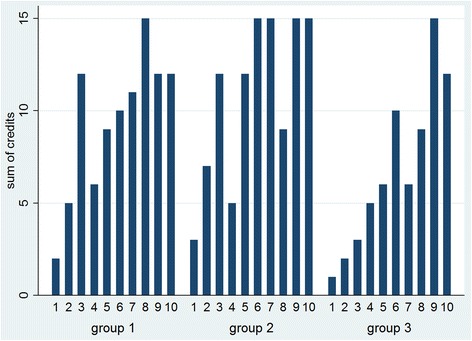
Evaluating supply management per group over 10 months: credits earned each month

In Fig. 3, the sum of the credits earned by each group in each month is showed, representing the availability and the accuracy of their stock cards, as well as the occurrence of stock-outs. The maximum number of credits that could be earned in each group was 15 per month (maximum 3 credits for each of the 5 health centres). In group 2 however, data from 1 health centre is missing for month 4, meaning that the group could only earn 12 credits that month.

Group 2 was the first group to obtain the maximum score: in month 6 all health centres could show the 6 stock cards without calculation mistakes and without reported stock-outs. With the exception of month 8, they continued to reach the maximum number of credits. Also in groups 1 and 3 an improvement in supply management is visible.

The monthly average number of credits earned was 9.4/15, 10.9/15 and 6.9/15 in group 1, 2 and 3 respectively. The difference between group 2 and 3 was borderline significant (*p* = 0.07).

Among the 15 health centres, credits measured differed significantly over time (Kendall’s coefficient = 0.24; *p* = 0.00). When looking at each of the groups separately, Kendall’s coefficient is smaller for the intervention groups (0.17 and 0.03 for group 1 and 2 respectively, versus 0.31 for group 3), but the difference over the 10 months is only significant in group 3 (*p* = 0.01).

##### Number of stock cards

**Fig. 4:**
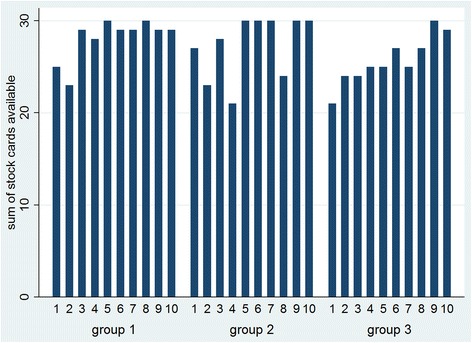
Stock cards of 6 family planning methods available per group over 10 months

A first requirement for the health centres to obtain credits was to have a stock card for each of the 6 contraceptive methods. Figure 4 shows the number of stock cards that each group presented during the 10 health facility assessments. The results resemble those of the entire evaluation system, i.e. the credits: all groups improved, and groups 1 and 2 seem to have done so more rapidly than the control group.

The stock card that was most often missing among all 15 health centres and over the 10 months was the card for females condoms (34/149), followed by the card for IUDs (24/149) and implants (18/149). Stock cards for both the low-dose and the combined contraceptive pill, and the injectable contraceptive were hardly ever missing (4/149, 2/149, 1/149 respectively). Especially in health centres in the control group, the stock card of female condoms continued to be lacking throughout the project (data not shown).

##### Percentage of stock cards with calculation mistakes

**Fig. 5:**
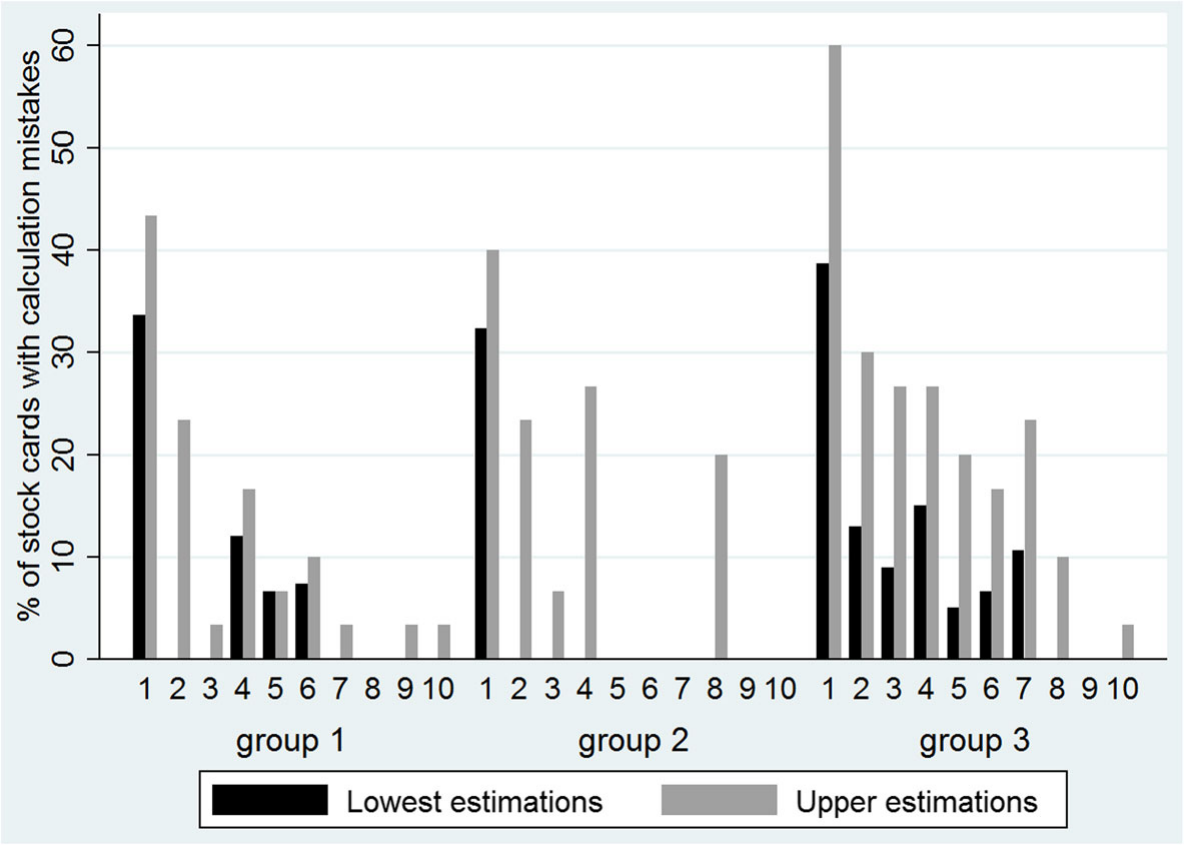
Percentage of stock cards with a calculation mistake per group over 10 months

Besides having a stock card for all contraceptives, health centres of groups 1 and 2 were required to fill them in accurately in order to earn a credit. Changes over time among the 3 groups are represented in Fig. 5. The results suggest that, in both analyses (lower and upper estimations), group 3 continued to have more stock cards with calculation mistakes compared to groups 1 and 2. Group 2 performed best in that we counted more months without any stock cards with calculation mistakes compared to groups 1 and 3.

##### Percentage of stock cards reporting a stock-out

**Fig. 6:**
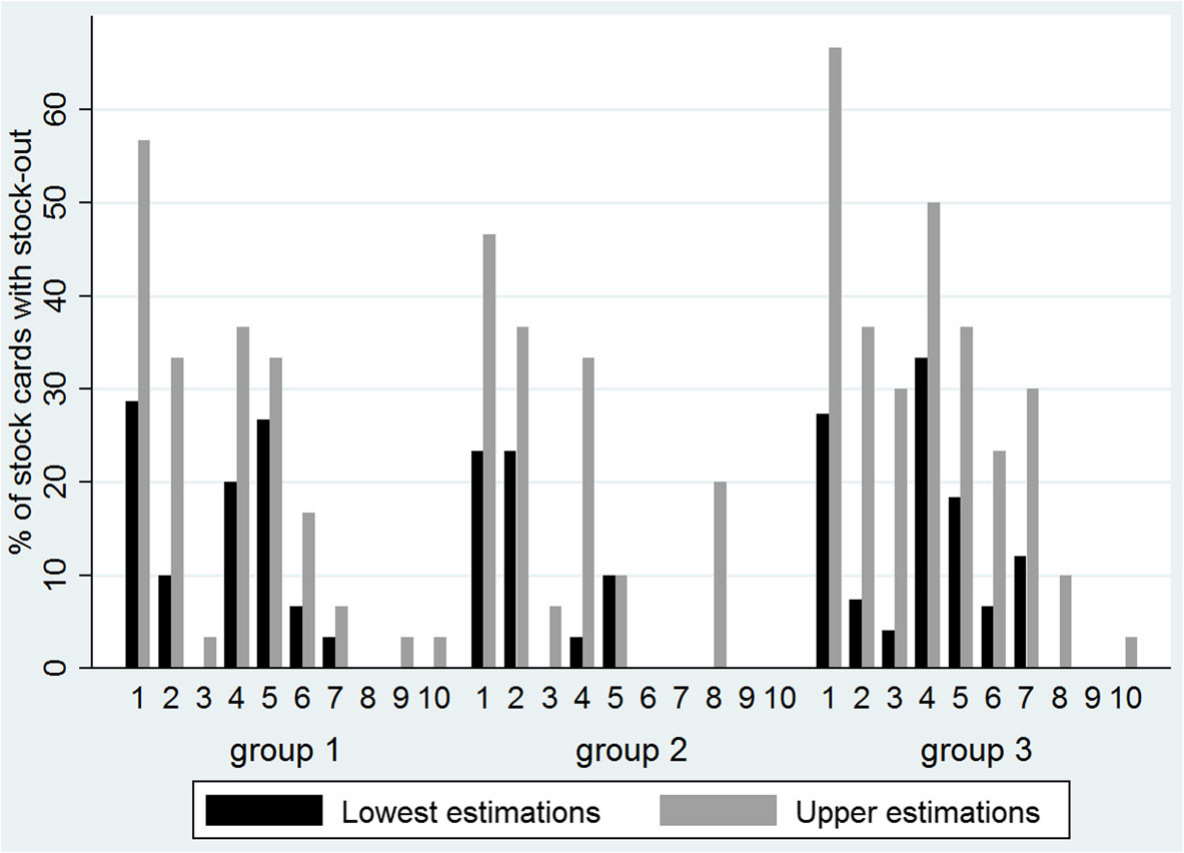
Percentage of stock cards reporting a stock-out per group over 10 months

A third point of evaluation was the number of stock cards reporting a stock-out. Group 2 practically eradicated stock-outs from the second half of the project onwards. Groups 1 and 3 eliminated stock-outs during the final 3 months but only in the analysis that does not consider a missing stock card as a stock-out (i.e. the lowest estimations). In the upper estimations, groups 1 and 3 continued to report stock-outs up until the end of the project (Fig. 6).

#### Stock-outs on the day of the assessment

**Fig. 7:**
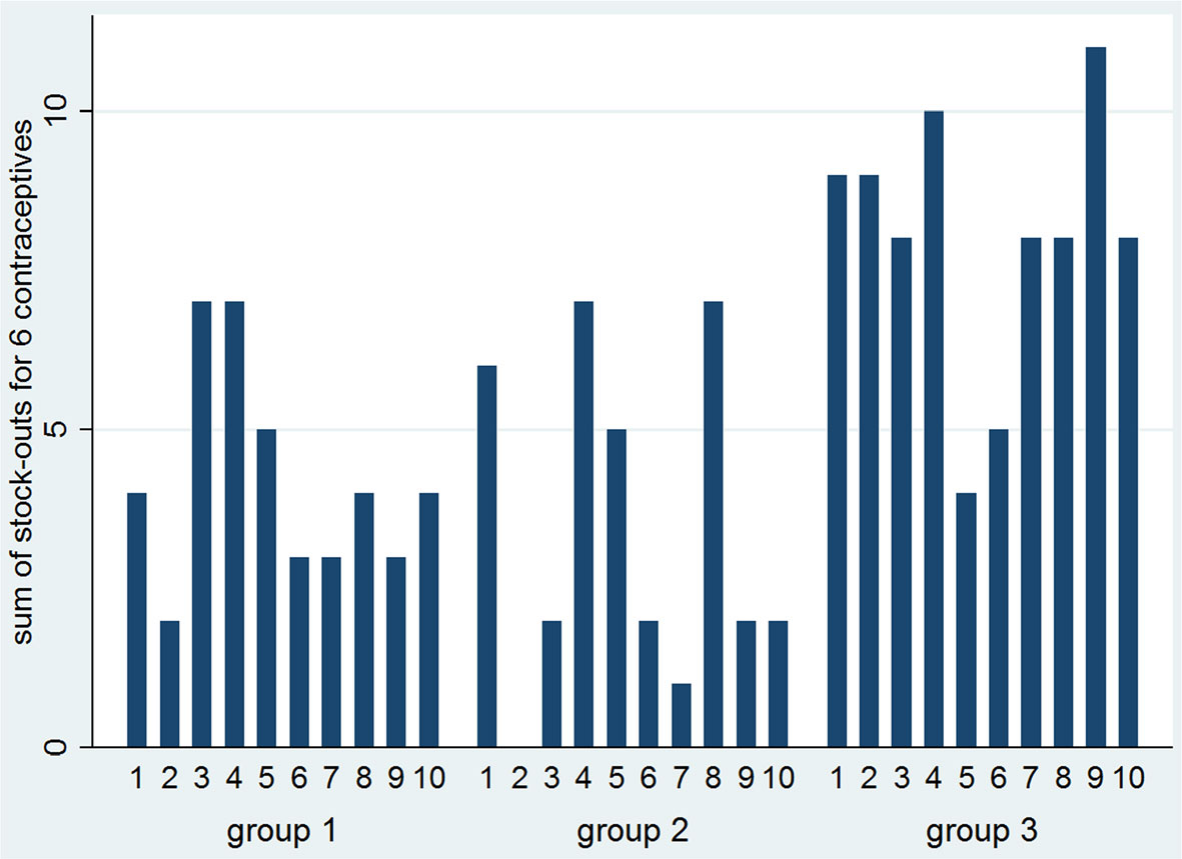
Number of stock-outs counted at the day of the assessment, per group over 10 months. Group 2: Only 4 centres were assessed during round 1 and 6, instead of 5

Actual stock of the 6 contraceptive methods was counted monthly during the health facility audits, enabling us to define ‘stock-outs on the day of the assessment’. Among the 15 health centres, during the 10 months intervention, 68 stock-outs of female condoms were counted, 32 for IUDs, 24 for implants, 18 for the injectable contraceptive, 9 for the combined pill and 5 for the low-dose pill (data not shown). In Fig. 7, an overview of counted stock-outs is presented per month per group. Given that each group contained 5 health centres and that 6 family planning methods were verified, the maximum number of stock-outs per month is 30. During most months, groups 1 and 2 had less than 5 stock-outs; group 3 on the other hand had often close to 10 stock-outs per month. The difference in the monthly average of number of stock-outs between the intervention groups and the control group was only significant for group 2 (*p* = 0.05; Additional file 3).

During the entire project, only group 2 had 1 month when no stock-outs occurred. This is in contrast with the results derived from the stock cards which showed that even according to the ‘upper estimations’ group 2 reached 4 months without reported stock-outs (Fig. 6).

**Fig. 8:**
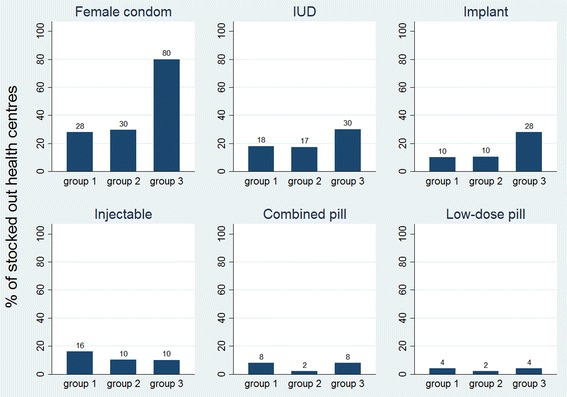
Percentage of stocked out health centres, per method per group over 10 months

Figure 8 shows the percentage of health centres stocked out on the day of assessment during the 10 months for each of the 6 contraceptive methods verified. The control group tended to have more stock outs of female condoms, IUDs and implants but not of the other contraceptive methods. The difference between the control group and the intervention groups is however only statistically significant for female condoms (Additional file 3). Overall, stock-outs occurred more for those methods that are less used in Mozambique (female condom, IUD and implants).

## Discussion

### Measuring motivation

We did not detect significant changes in motivation over time even though supply management did improve in all health centres, which might suggest that there is no relation between motivation and stock monitoring. However, the literature does provide evidence for the link between motivation and work performance [13] hence we might have been unable to demonstrate this relation. Two main reasons could be behind this: 1) a larger sample size is needed to detect changes, and 2) participants over-reported their motivation, especially at baseline. Since motivation cannot be measured in a direct way, it can only be derived from behaviour (e.g. absenteeism) or from questioning individuals on their perceptions regarding motivational outcomes, which is what the questionnaire we used does. An important remark however, is that the questionnaire was administered through a face-to-face interview, which may have resulted in more positive answers than would have been the case with self-administered questionnaires [7]. Qualitative interviews can help to better understand levels of motivation, however, if looking for quantitative estimations, studies might want to focus on only one component of motivation, such as burnout, in order to increase the validity of the measured item [19].

### Measuring stock-outs

Stock-outs reported by the stock cards and stock-outs identified by counting stock did not always lead to the same results. According to the first method, several health centres had months without stock-outs while the stock-counting data did not confirm this (Fig. 6 versus Fig. 7). One explanation is that the stock cards were not filled in correctly. Besides this ‘under-reporting’, it might also be that the counted stock had errors, if for example supply is spread among the facility and not kept in the pharmacy, where stock was mainly counted. Both measurements clearly have their limitations and researchers should carefully choose their data collection method of preference depending on the aim of the study and the context in which it is carried out.

While there is some discrepancy between the results of the 2 stock-out measurements, the data also show similarity: stocks cards were mostly missing for female condoms, IUDs and implants, which are also the methods that were mostly stocked out according to the count data. Also the number of stock-outs appeared to be lower in groups 1 and 2 compared with the control group according to the 2 measurements of stock-outs. However, estimating accurately the occurrence of stock-outs and especially the duration of each stock-out remains difficult in settings where stock is poorly registered. Better storage and electronic registration systems would not only improve supply management significantly but would also give more insight in the causes and frequency of stock-outs.

### Improving motivation and supply management

#### Reasons behind stock-outs

Comparing the usage of contraceptive methods in Mozambique with the observed stock-outs of each method, we counted more stock-outs for methods that are less used (female condoms, implants and IUDs) as opposed to the more popular methods such as the injectable and the pill [1]. More in-depth research is necessary to verify whether this difference is widespread and to identify the associations between stock-outs, demand and uptake (e.g. through mediation analysis and/or qualitative research). Definitely the fact that the stock card of female condoms was very often missing and that these stock-outs occurred significantly more in the control group compared with the intervention groups calls for extra attention: research has shown that acceptability goes hand in hand with having access and experience with the device [20], meaning that stock-outs hampering exposure and familiarization might directly influence the demand.

Reasons behind these stock-outs thus need to be investigated in order to identify potential solutions that could optimize the usage of these methods. Stock-outs can occur due to shortages at the level of the warehouse, or other logistical problems, but health workers might also order less of a certain contraceptive for various reasons. In Tanzania, for example, providers reported that they were not always properly trained to offer all methods or to address misconceptions [2]. Some providers also just might perceive a method as unpopular and hence stop ordering it, or may have personal preferences for only providing certain methods. The frequent lack of female condoms and poor supply management of implants and IUDs in some health centres assessed in this study, suggest indeed that some facilities have problems inherent with certain methods, as opposed to those facilities where stock-outs occur occasionally because of more temporary problems such as peaks in demand during national health weeks or inaccessible roads during the rainy season. As such, an understanding of different types of stock-outs – continuous versus occasional – could enable the identification of appropriate solutions in different situations. A push approach, rather than the current pull procurement system, could for example be a first step to tackle the problem of continuous stock-outs since it could encourage providers to start introducing methods that are now less used.

#### Need for supportive supervision

While stock-outs were recurrent even during the interventions, the data suggest an improvement in stock management among all 3 groups, including the control group (Fig. 3 – significant Kendall’s coefficient of 0.24 and 0.31 for all health centres and for those in group 3, respectively). The monthly facility audits, although initially not considered as an intervention but rather a data collection activity, may have induced a downward trend in terms of missing stock-cards, calculation errors and reported stock-outs on the cards of the health centres of both intervention and control groups. Frequent supervision and quality control have been recognized as important and effective in improving performance of health staff [21–23]. It is likely that the audits were perceived as a form of recognition of the staff’s efforts and achievements. Participants described indeed these visits as motivating and supportive during wrap-up discussions. Given that our monthly visits consisted of simple inventory checking and can be built into the supervision visits that are already integrated in the health system, it is recommended that the Ministry of Health in Mozambique emphasizes the need and importance of the foreseen supervision visits so as to ensure that they happen frequently and provide the guidance and encouragement health providers need. In addition, more longitudinal research is needed to truly verify the impact of such ongoing training and on-the-spot support in other settings.

#### Impact of evaluation reports and material incentives

Compared with group 3, supply management seemed to improve more in groups 1 and 2 (Fig. 3), indicating the possibility that the distribution of evaluation reports was also valued, as well as the material incentives for group 2. It is notable that monthly average results from group 2, i.e. the number of credits and the counted stock-outs, were significantly different from the control group. With this we provide further support to implement programmes focussing on intrinsic motivation by offering non-financial incentives [10] so as to confirm these findings and to estimate and understand the role of such incentives for motivation of health care providers. Further research is also needed to assess the relative weight of the different types of intervention, in terms of their impact on motivation and performance.

### Limitations

The exclusion of 6 health centres and a lower participation rate than expected led to a small sample which affected the power of the study. In addition, less motivated health staff might have refused to participate and, together with potentially socially desirable answers given by those who did enrol, this might have led to an overestimation of the reported motivation with limited room to detect improvements as a result of the interventions. Likewise, the small sample made it impossible to apply more in-depth analysis to verify changes and thus the potential impact of the interventions on supply management. Wilcoxon Mann–Whitney tests were carried out to compare the monthly average scores of the intervention groups with those from the control group, and Kendall’s coefficients were used to verify the difference between the monthly measurements, however time series analysis should be carried out, on larger samples, as to verify trends or changes in time. Given the descriptive nature of this study, the external validity is limited.

## Conclusions

Even though our project was a small-scale prospective study in a limited region in Maputo Province, Mozambique, it has highlighted some important issues with regard to motivation and supply management. We found indications that while evaluation reports and material incentives might have the potential to improve health care providers’ motivation and supply management, supportive supervision in the form of regular audits providing follow-up and feedback is probably also key, possibly because it enhances intrinsic motivation. Special attention should go to preventing stock-outs of family planning methods that are less used, given that these include the highly effective longer-acting methods, and that these stock-outs could be contributing to the lower demand.

## Additional files

Additional file 1: Motivation – differences among the 3 groups. (DOCX 22 kb)

Additional file 2: Motivation – changes over time. (DOCX 24 kb)

Additional file 3: Stocked out health centres for each of the family planning methods, averaged over the 10 month intervention period. (DOCX 17 kb)
