# Supplementary material for: The impact of facility audits, evaluation reports and incentives on motivation and supply management among family planning service providers: an interventional study in two districts in Maputo Province, Mozambique
Source: BMC Health Serv Res. 2017 May 2;17:313. doi: 10.1186/s12913-017-2222-3 (PMC5414138; doi:10.1186/s12913-017-2222-3)
Supplement: Supplementary file 1 — Motivation – differences among the 3 groups. (DOCX 22 kb) [file 12913_2017_2222_MOESM1_ESM.docx]

ADDITIONAL FILE 1

**Motivation – differences among the 3 groups**

In the following 3 tables (1.1 – 1.3), motivation measured at baseline and during the 1^st^ and 2^nd^ follow-up is compared among the 3 groups. Using the Kruskal Wallis test, the total motivation as well as the subcomponents of the motivational scale are compared. For none of the measurements, a statistical difference was detected. This means that at no point in time and for none of the measured components the groups differed significantly in motivation.

**Table 1.1: Motivation reported by health care providers at baseline**

|  | **Group 1 (n=10)** | **Group 2 (n=12)** | **Group 3 (n=17)** | **Kruskal Wallis** |
| --- | --- | --- | --- | --- |
|  | **Median (IQR)** | **Median (IQR)** | **Median (IQR)** | **p-value** |
| **OVERALL MOTIVATION (max 105)** | **88.5 (87-92)** | **84.5 (79-93)** | **93 (86.5-95)** | **0.23** |
| General motivation | 3.7 (3.0-4.3) | 3.8 (3.5-4.2) | 3.7 (3.3-4.3) | 0.91 |
| Burn out (reversed) | 3.5 (3.0-4.5) | 3.3 (2.5-4.3) | 3.5 (3.0-4.0) | 0.65 |
| Job satisfaction | 4.7 (4.3-5.0) | 4.3 (3.7-4.8) | 4.3 (3.7-5.0) | 0.37 |
| Intrinsic motivation | 4.7 (4.3-4.7) | 4.2 (3.8-5.0) | 4.7 (4.7-5.0) | 0.18 |
| Organizational commitment | 4.2 (4.2-4.4) | 3.9 (3.2-4.4) | 4.4 (3.8-4.8) | 0.37 |
| Conscientiousness | 5.0 (4.0-5.0) | 5.0 (4.3-5.0) | 5.0 (4.5-5.0) | 0.93 |
| Timeliness and attendance | 4.3 (4.0-4.7) | 4.5 (4.0-5.0) | 4.7 (4.0-5.0) | 0.64 |

**Table 1.2: Motivation reported by health care providers at 1^st^ follow-up**

|  | **Group 1 (n=10)** | **Group 2 (n=12)** | **Group 3 (n=17)** | **Kruskal Wallis** |
| --- | --- | --- | --- | --- |
|  | **Median (IQR)** | **Median (IQR)** | **Median (IQR)** | **p-value** |
| **OVERALL MOTIVATION (max 105)** | **87 (83-88)** | **90 (86-93)** | **86 (83-91)** | **0.69** |
| General motivation | 3.7 (2.7-4.0) | 3.7 (3.3-4.0) | 3.3 (3.3-4.0) | 0.92 |
| Burn out (reversed) | 3.0 (3.0-4.0) | 4.5 (2.5-4.5) | 3.0 (2.0-4.0) | 0.66 |
| Job satisfaction | 4.0 (4.0-5.0) | 4.3 (4.0-4.7) | 4.3 (4.3-4.7) | 0.98 |
| Intrinsic motivation | 4.7 (4.3-4.7) | 4.5 (3.7-5.0) | 4.3 (4.0-5.0) | 0.65 |
| Organizational commitment | 4.0 (3.8-4.6) | 4.0 (4.0-4.2) | 4.0 (3.6-4.6) | 0.99 |
| Conscientiousness | 4.5 (4.5-5.0) | 5.0 (4.5-5.0) | 5.0 (4.5-5.0) | 0.27 |
| Timeliness and attendance | 4.7 (4.3-4.7) | 5.0 (4.7-5.0) | 4.7 (4.7-5.0) | 0.17 |

| **Table 1.3: Motivation reported by health care providers at 2^nd^ follow-up;** | **Group 1 (n=10)** | **Group 2 (n=12)** | **Group 3 (n=17)** | **Kruskal Wallis** |
| --- | --- | --- | --- | --- |
|  | **Median (IQR)** | **Median (IQR)** | **Median (IQR)** | **p-value** |
| **OVERALL MOTIVATION (max 105)** | **90 (88-90)** | **87 (83-90)** | **87 (83-90)** | **0.24** |
| General motivation | 4.0 (3.3-4.3) | 3.8 (3.3-4.3) | 3.7 (3.3-4.0) | 0.89 |
| Burn out (reversed) | 3.5 (3.0-4.0) | 3.0 (3.0-4.5) | 4.0 (2.5-4.5) | 0.68 |
| Job satisfaction | 4.3 (4.3-4.7) | 4.8 (4.3-5.0) | 4.7 (3.7-4.7) | 0.12 |
| Intrinsic motivation | 4.7 (4.3-5.0) | 4.3 (4.0-4.3) | 4.7 (4.3-4.7) | 0.15 |
| Organizational commitment | 4.1 (4.0-4.4) | 4.0 (3.8-4.3) | 4.0 (3.6-4.4) | 0.70 |
| Conscientiousness | 4.8 (4.5-5.0) | 5.0 (4.5-5.0) | 5.0 (4.5-5.0) | 0.87 |
| Timeliness and attendance | 4.7 (4.3-5.0) | 4.2 (4.0-4.8) | 4.7 (4.3-5.0) | 0.44 |
