# Supplementary material for: The impact of facility audits, evaluation reports and incentives on motivation and supply management among family planning service providers: an interventional study in two districts in Maputo Province, Mozambique
Source: BMC Health Serv Res. 2017 May 2;17:313. doi: 10.1186/s12913-017-2222-3 (PMC5414138; doi:10.1186/s12913-017-2222-3)
Supplement: Supplementary file 2 — Motivation – changes over time. (DOCX 24 kb) [file 12913_2017_2222_MOESM2_ESM.docx]

ADDITIONAL FILE 2

**Motivation – changes over time**

In table 1.4, motivation measured at baseline and during the 1^st^ and 2^nd^ follow-up is presented. Using the Wilcoxon signed ranks test, the total motivation as well as the subcomponents and the individual questions are compared: for the entire cohort of participants, baseline results were compared with the results of the 1st follow-up, as well as with the results of the 2^nd^ follow-up. As such, changes in time could be verified. For few measurements, a statistical difference was detected, but no clear trend could be detected. This means that in general, motivation, reported by the participants, did not change in time.

**Table 1.4: Comparing baseline motivation with motivation reported at 1^st^ and 2^nd^ follow-up**

| **MOTIVATION PROVIDERS:** | **Baseline (n=39)** | **1^st^ Follow-up (n=39)** | **2^nd^ Follow-up (n=39)** | **Wilcoxon signed ranks test (baseline – 1^st^ follow-up)** | **Difference 1^st^ follow-up -baseline** | **Wilcoxon signed ranks test (baseline – 2^nd^ follow-up)** | **Difference 2^nd^ follow-up -baseline** |
| --- | --- | --- | --- | --- | --- | --- | --- |
|  | **Median (IQR)** | **Median (IQR)** | **Median (IQR)** | **p-value** | **IQR**** | **p-value** | **IQR**** |
| **OVERALL MOTIVATION (max 105)** | **89 (84-94)** | **88 (83-91.5)** | **87.5 (85-90)** | **0.97** | **-6 – 5** | **0.89** | **-6 – 4** |
| **General motivation** | **3.7 (3.3-4.3)** | **3.7 (3.3-4.0)** | **3.7 (3.3-4.3)** | **0.80** | **-0.7 – 0.3** | **0.86** | **-0.7 – 0.7** |
| Feel motivated to work hard | 4 (3-5) | 4 (4-5) | 4 (2-4) | 0.98 | -1 – 1 | 0.39 | -1 – 0 |
| Only do this job to get paid* | 5 (4-5) | 5 (4-5) | 4 (4-5) | 0.46 | 0 – 1 | 0.12 | -1 – 0 |
| Do this job to have long-term security | 3 (2-4) | 2 (2-4) | 4 (2-4) | 0.39 | -1.5 – 1 | 0.08 | 0 – 2 |
| **Burn out (reversed)** | **3.5 (3.0-4.5)** | **3.0 (3.0-4.0)** | **3.5 (3.0-4.5)** | **0.32** | **-1 – 0.5** | **0.99** | **-0.5 – 1** |
| Feel emotionally drained at end of day* | 4 (2-4) | 2 (2-4) | 4 (2-4) | 0.02 | -2 – 0 | 0.14 | -2 – 0 |
| At times, you dread facing a day at work* | 4 (2-5) | 4 (3-5) | 4 (4-5) | 0.75 | -1 – 1 | 0.40 | -1 – 1 |
| **Job satisfaction** | **4.3 (4.0-5.0)** | **4.3 (4.0-4.7)** | **4.3 (4.0-5.0)** | **0.32** | **-0.3 – 0.7** | **0.54** | **-0.3 – 0.3** |
| Overall, I am very satisfied with my job | 5 (4-5) | 5 (5-5) | 5 (4-5) | 0.70 | 0 – 0.5 | 0.61 | 0 – 0 |
| Not satisfied with my colleagues* | 4 (4-5) | 4 (4-5) | 4(4-5) | 0.29 | 0 – 1 | 0.30 | -1 – 1 |
| I am satisfied with my supervisor | 4 (4-5) | 4 (4-5) | 4 (4-5) | 0.23 | 0 – 1 | 0.93 | -1 – 1 |
| **Intrinsic motivation** | **4.7 (4.0-5.0)** | **4.3 (4.0-5.0)** | **4.3 (4.0-4.7)** | **0.37** | **-0.5 – 0.3** | **0.38** | **-0.7 – 0.3** |
| Satisfied with opportunity to use abilities | 5 (4-5) | 5 (4-5) | 5 (4-5) | 0.46 | -1 – 0 | 0.09 | -1 – 0 |
| Satisfied with accomplishing something | 5 (4-5) | 5 (4-5) | 4 (4-5) | 0.88 | -.5 – 0 | 0.08 | -1 – 0 |
| My work is not valuable these days* | 4 (4-5) | 4 (4-5) | 4 (4-5) | 0.36 | -1 – 0 | 0.56 | -1 – 1 |
| **Organizational commitment** | **4.2 (3.6-4.6)** | **4.0 (3.8-4.6)** | **4.0 (3.8-4.4)** | **0.97** | **-0.4 – 0.4** | **0.38** | **-0.4 – 0.2** |
| Proud to work for this health facility | 4 (4-5) | 5 (4-5) | 4 (4-5) | 0.87 | 0 – 0 | 0.58 | 0 – 0 |
| My values and this facility’s are similar | 4 (4-5) | 4 (4-4) | 4 (4-4) | 0.05 | -1 – 0 | 0.32 | -1 – 0 |
| Glad to work for this facility | 4 (2-5) | 4 (2-4) | 4 (2-4) | 0.79 | -1 – 1 | 0.53 | -1 – 0 |
| Feel little commitment to this facility* | 4 (4-5) | 4 (4-5) | 4 (4-5) | 0.36 | 0 – 1 | 0.22 | -1 – 0 |
| This facility inspires me to do my best | 5 (4-5) | 5 (4-5) | 4 (4-5) | 0.54 | 0 – 0 | 0.79 | -1 – 1 |
| **Conscientiousness** | **5 (4.5-5.0)** | **5 (4.5-5.0)** | **5 (4.5-5.0)** | **0.96** | **0 – 0** | **0.84** | **0 - 0** |
| I am a hard worker | 5 (4-5) | 5 (4-5) | 5 (4-5) | 0.56 | 0 – 0 | 0.62 | 0 – 0 |
| Do things without being asked or told | 5 (5-5) | 5 (5-5) | 5 (5-5) | 0.16 | 0 – 0 | 0.02 | 0 – 0 |
| **Timeliness and attendance** | **4.7 (4.0-5.0)** | **4.7 (4.7-5.0)** | **4.7 (4.0-5.0)** | **0.01** | **0 – 0.7** | **0.72** | **-0.3 – 0.3** |
| I am punctual about coming to work | 5 (4-5) | 5 (4-5) | 5 (4-5) | 0.03 | 0 – 0 | 0.05 | 0 – 1 |
| I am often absent from work | 5 (4-5) | 5 (4-5) | 5 (4-5) | 0.15 | 0 – 1 | 0.80 | -1 – 0 |
| Not a problem if I sometimes come late* | 5 (4-5) | 5 (5-5) | 5 (4-5) | 0.06 | 0 – 1 | 0.33 | -1 – 0 |

*reversed score: the higher the score, the higher the motivation

** inter-quartile rang
