# Supplementary material for: The impact of facility audits, evaluation reports and incentives on motivation and supply management among family planning service providers: an interventional study in two districts in Maputo Province, Mozambique
Source: BMC Health Serv Res. 2017 May 2;17:313. doi: 10.1186/s12913-017-2222-3 (PMC5414138; doi:10.1186/s12913-017-2222-3)
Supplement: Supplementary file 3 — Stocked out health centres for each of the family planning methods, averaged over the 10 month intervention period. (DOCX 17 kb) [file 12913_2017_2222_MOESM3_ESM.docx]

ADDITIONAL FILE 3

**Stocked out health centres for each of the family planning methods, averaged over the 10 month intervention period**

In table 2.1, the average number of stock-outs per family planning method over 10 months is presented. The stock-outs are defined by counting stock on the day of the facility audits. Additionally, results are also expressed as the percentage of health facilities stocked out for each of the contraceptives during the intervention^[[1]](#footnote-1)^. Using the Wilcoxon Mann-Whitney test, the number of stock-outs among the groups are compared for each of the family planning methods. Only for the number of stock-outs of female condoms a statistical difference was detected (i.e. the p-value was always lower than 0.05). This means that health centres in the control group had more female condom stock-outs during the intervention period compared with the 2 intervention groups.

**Table 2.1: Average number of stocked out health centres for each method per group (over 10 months);**

**comparing results of group 1 and group 2 versus the control group by use of a Wilcoxon-Mann Whitney test**

| Method | # of assessed HC that were stocked out of the FP method at day of the facility assessment | | | Wilcoxon-Mann Whitney test (group x vs control) - p-value | |
| --- | --- | --- | --- | --- | --- |
|  | Group1 | Group2 | Control | Group1 | Group2 |
| Female condom | 1.4/5 | 1.5/5 | 4.0/5 | 0.03 | 0.02 |
| IUD | 0.9/5 | 0.9/5 | 1.5/5 | 0.74 | 0.50 |
| Implant | 0.5/5 | 0.5/5 | 1.4/5 | 0.26 | 0.46 |
| Injectable - Depo. | 0.8/5 | 0.5/5 | 0.5/5 | 0.52 | 1.00 |
| Pill - Microlut | 0.2/5 | 0.1/5 | 0.2/5 | 1.00 | 0.51 |
| Pill - Microgynon | 0.4/5 | 0.1/5 | 0.4/5 | 1.00 | 0.19 |

1. [↑](#footnote-ref-1)
